# Extracellular vesicles from pasteurized *Akkermansia muciniphila* ameliorate inflammatory bowel disease through suppression of STING-driven inflammatory signaling

**DOI:** 10.3389/fmicb.2026.1876636

**Published:** 2026-07-22

**Authors:** Ling Zou, Zhilong Jia, Yutong Shu, Xiang You, Jingjing Ma, Yu Wang, Jinhan He, Yining Xu

**Affiliations:** 1 Department of Pharmacy, Institute of Metabolic Diseases and Pharmacotherapy, West China Hospital, Sichuan University, Chengdu, China; 2 Key Laboratory of Drug-Targeting and Drug Delivery System of the Education Ministry and Sichuan Province, West China School of Pharmacy, Sichuan University, Chengdu, China

**Keywords:** *Akkermansia muciniphila*, extracellular vesicles, gut microbiota, inflammatory bowel disease, pasteurized, STING/IκB/NF-κB signaling axis

## Abstract

**Introduction:**

*Akkermansia muciniphila* (*A. muciniphila*) has shown considerable potential in maintaining intestinal barrier homeostasis and regulating host inflammatory responses, both of which are commonly disrupted in inflammatory bowel disease (IBD). However, the therapeutic application of live *A. muciniphila* in IBD remains controversial. Interestingly, *A. muciniphila*-derived extracellular vesicles (AEVs) have been reported to improve intestinal barrier function, immune status, and gut microbiota composition, and may exert superior efficacy in IBD. In parallel, pasteurized *A. muciniphila* has been shown to retain, or even enhance, beneficial bioactivity compared with the live bacterium in certain disease settings. Here, we investigated whether extracellular vesicles derived from pasteurized *A. muciniphila* (PAEVs) preserve or further enhance the anti-inflammatory and barrier-protective effects of the parental bacterium.

**Methods:**

A dextran sulfate sodium (DSS)-induced mouse model of colitis was used to evaluate the therapeutic effects of PAEVs and AEVs. Disease severity, body weight loss, colonic histopathology, inflammatory cytokine expression, intestinal barrier integrity, inflammatory signaling pathways, and gut microbiota composition were assessed.

**Results:**

PAEVs markedly attenuated DSS-induced colitis, as evidenced by reduced weight loss, improved colonic histology, decreased levels of TNF-*α*, IL-6, and IFN-*γ*, and enhanced tight junction proteins. By contrast, AEVs improved only limited parameters, including Occludin expression and TNF-*α* levels. Mechanistically, PAEV-mediated protection may be associated with suppression of the STING/IκB/NF-κB signaling axis and remodeling of the gut microbiota.

**Discussion:**

These findings indicate that PAEVs effectively alleviate experimental IBD by enhancing tight junction proteins, suppressing some inflammatory cytokines, and modulating gut microbiota composition. Compared with AEVs, PAEVs exhibit broader protective effects, suggesting that extracellular vesicles derived from pasteurized *A. muciniphila* may represent a promising postbiotic strategy for IBD intervention. Importantly, this study offers the first systematic comparison of extracellular vesicles derived from live and pasteurized *A. muciniphila*, highlighting PAEVs as a distinct and potentially more effective postbiotic vesicle formulation for IBD intervention.

## 1 Introduction

Inflammatory bowel disease (IBD) is a chronic, relapsing inflammatory disorder of the gastrointestinal tract with a not yet fully understood etiology (Segal et al., 2021). IBD has emerged as a global public health concern, with its incidence and prevalence continuing to rise. This disease not only markedly impairs patients’ quality of life, but also imposes a substantial burden on families and healthcare systems (Kaplan, 2025). Currently, the clinical management of IBD mainly relies on aminosalicylates, glucocorticoids, immunosuppressants, and biologic agents (Appiah et al., 2025). Although these therapies can, to some extent, control acute inflammation, alleviate clinical symptoms, and maintain disease remission, their use is still limited by significant adverse effects associated with long-term administration, as well as primary non-response or secondary loss of response in some patients (Singh et al., 2024). These limitations, at least in part, reflect the complexity and multifactorial nature of IBD pathogenesis. Therefore, the development of safe and effective therapeutic strategies capable of simultaneously targeting multiple pathogenic processes, including inflammatory responses, mucosal barrier dysfunction, and gut microbiota dysbiosis, is of great clinical importance.

Existing research indicates that the occurrence and development of IBD are closely related to the destruction of the intestinal mucosal barrier, persistent immune-inflammatory activation, and dysbiosis of the gut microbiota (Iacucci et al., 2024). In this context, intervention strategies targeting the gut microbiota and its functional products have received widespread attention. *Akkermansia muciniphila* (*A. muciniphila*) is a symbiotic bacterium that colonizes the intestinal mucus layer and has been regarded in recent years as a highly promising next-generation probiotic. An increasing number of studies have shown that *A. muciniphila* plays an important role in maintaining the integrity of the mucus layer, enhancing the intestinal epithelial barrier, regulating host metabolism, and inhibiting inflammatory responses (Mo et al., 2024). However, its therapeutic role in IBD exhibits a contradictory effect. Some studies have shown that *A. muciniphila* can significantly improve symptoms of dextran sulfate sodium salt (DSS)-induced acute colitis, repair the intestinal barrier, and reduce the levels of inflammatory factors (Qu et al., 2021; Panzetta and Valdivia, 2024). However, some studies have found that under certain genetic susceptibility backgrounds, *A. muciniphila* may exhibit pro-inflammatory effects. For example, in susceptible models such as *Il10*^*−*/−,^ its abnormal enrichment is associated with exacerbation of IBD (Seregin et al., 2017). The possible reason may be that the overcolonization or abnormal enrichment of live *A. muciniphila* disrupts the dynamic balance between mucus secretion and degradation, leading to a thinning of the mucus layer, increased intestinal permeability, and downregulation of tight junction protein expression, thereby further amplifying the local inflammatory response (Seregin et al., 2017; Qu et al., 2023). Therefore, in addition to the live bacteria themselves, many studies focus on the functional components derived from *A. muciniphila*, such as some related proteins and extracellular vesicles derived from *A. muciniphila* (AEVs). AEV, which remained the main fraction of *A. muciniphila*, could regulate immune responses, maintain barrier homeostasis, and influence gut microbiota in many diseases, such as IBD (Zheng et al., 2023; Abraham et al., 2025; Hong et al., 2025; Wu et al., 2025; Chen et al., 2026). However, some studies suggest that AEVs are not entirely inert anti-inflammatory components; under specific *in vitro* or disease contexts, they can induce IL-6 secretion, promote M1 macrophage polarization, and enhance the expression of inflammation-related molecules such as IFN-*γ* and Interleukin-1β (IL-1β), indicating a certain potential for pro-inflammatory immune activation (Kang et al., 2013). This suggests that AEV, while retaining anti-inflammatory activity, may also retain some pro-inflammatory components.

Pasteurization effectively inactivates microorganisms by denaturing bacterial enzymes (Zhu et al., 2025). Notably, probiotics like *A. muciniphila* pasteurized at 70 °C for 30 min may still retain beneficial effects (Plovier et al., 2017). Outer membrane protein of *A. muciniphila*, such as Amuc_1100, is stable at pasteurization temperatures (Plovier et al., 2017), suggesting that some of the probiotic effects of this bacterium may be mediated by heat-stable cellular components. More importantly, pasteurized *A. muciniphila* has shown good safety in human studies, with high safety and good tolerance observed in both animal experiments and human clinical trials (NCT02637115, NCT05348642). Interestingly, recent reports have also indicated that in DSS-induced colitis models, pasteurized *A. muciniphila* may outperform live bacteria in improving colitis, restoring microbial balance, and regulating metabolism (Han et al., 2025). This suggests that pasteurized *A. muciniphila* may also retain active components related to the treatment of IBD. Taken together, previous findings suggest that both *A. muciniphila*-derived EVs and pasteurized *A. muciniphila* may exert therapeutic effects in IBD. However, there is currently no systematic study evaluating the impact of other components of pasteurized *A. muciniphila* on IBD. Therefore, a thorough investigation into the roles and potential mechanisms of its different functional components in IBD will help further clarify the material basis of its therapeutic effects and provide a theoretical foundation for the development of related microbiome-based preparations.

**Figure 1:**
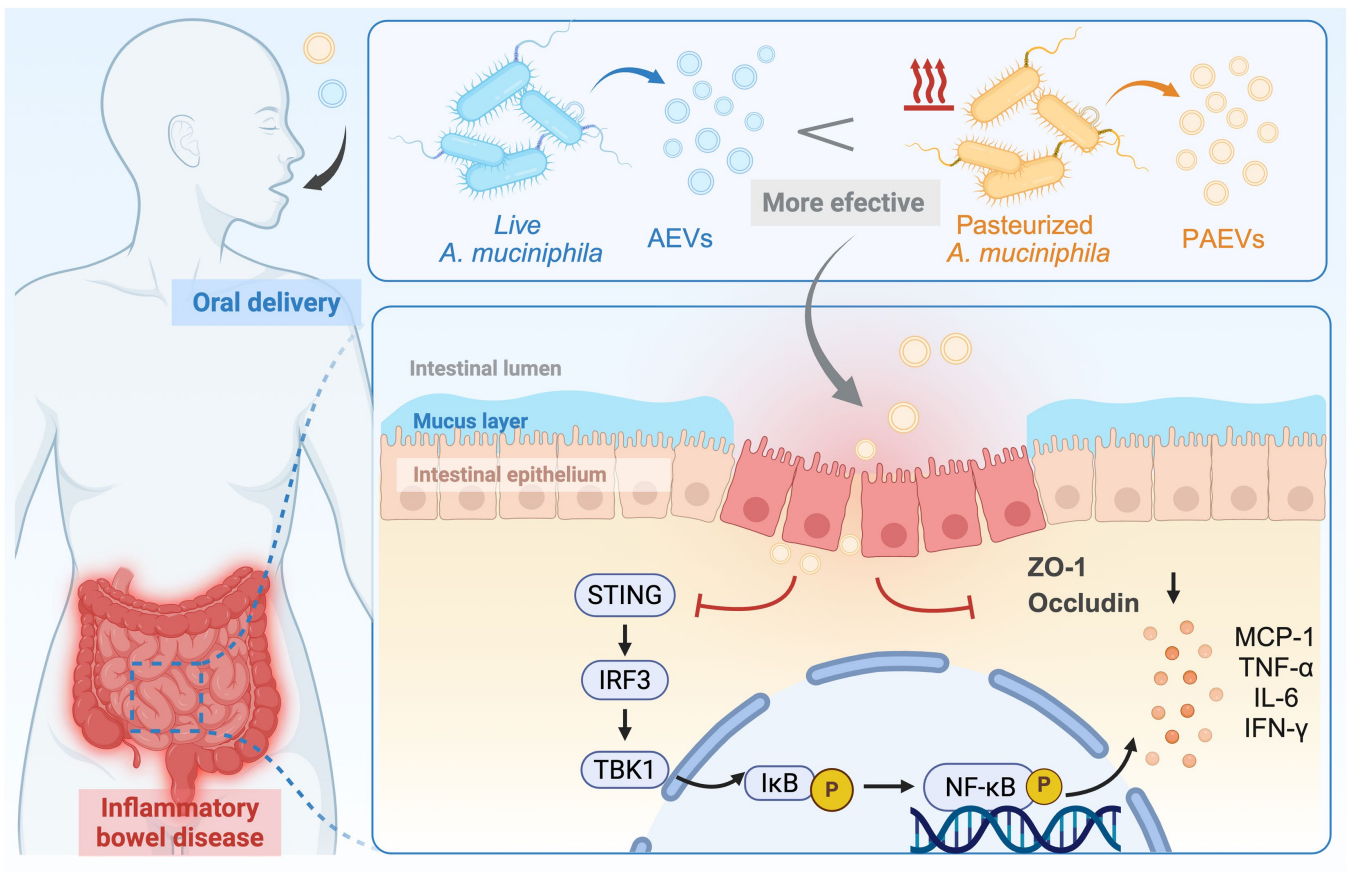
Schematic illustration of the potential mechanisms by which extracellular vesicles derived from pasteurized *A. muciniphila* are likely associated with anti-inflammatory effects involving suppression of the STING/IκB/NF-κB pathway, enhancement of tight junction proteins, and modulation of gut microbiota homeostasis. Created in BioRender. Xu, Y. (2026) https://BioRender.com/kkj1rla.

Although the beneficial effects of both live or pasteurized *A. muciniphila*, as well as naturally derived *A. muciniphila* extracellular vesicles, have been reported, it remains unclear whether pasteurization functionally alters these vesicles and if extracellular vesicles from pasteurized *A. muciniphila* exert superior therapeutic effects in IBD. This raises an important scientific question of whether the extracellular vesicles released or retained by *A. muciniphila* following pasteurization still maintain anti-inflammatory and barrier-protective properties, or might they even demonstrate enhanced biological activity. To address this, we isolated natural extracellular vesicles from the culture supernatant of live *A. muciniphila* (AEVs) and extracellular vesicles from the culture supernatant of pasteurized *A. muciniphila* (PAEVs), and compared their biological functions. The novelty of our study lies in the direct comparative analysis of AEVs and PAEVs regarding vesicle characterization, therapeutic efficacy, inflammatory signaling, epithelial barrier protection, and gut microbiota modulation. As shown in Figure 1, our study found that in a DSS-induced IBD mouse model, PAEV markedly reduced the severity of IBD, as evidenced by weight loss, improved colonic tissue pathology, decreased expression of selected pro-inflammatory cytokines including tumor necrosis factor-alpha (TNF-*α*), Interleukin-6 (IL-6), and Interferon-gamma (IFN-*γ*), and upregulation of tight junction proteins. It is worth noting that AEVs also exerted a protective effect; however, this effect was markedly weaker than that observed for PAEVs. Mechanistically, the protective effect mediated by PAEV is associated with suppression of the STING/IκB/NF-κB signaling axis. Collectively, these findings identify PAEVs as a distinct postbiotic vesicle preparation that provides broader protection against experimental IBD than conventional AEVs, thereby offering new theoretical support for the development of *A. muciniphila*-derived postbiotics for IBD intervention (Figure 1).

## 2 Methods

### 2.1 Preparation of the EVs and pasteurized EVs

The *A. muciniphila* strain was purchased from the American Type Culture Collection (ATCC, BAA-835). The strain was cultured in pre-reduced Brain-Heart Infusion (BHI) medium, supplemented with 0.5% (w/v) porcine gastric mucin (Orileaf, S12066-100 g), 0.05% (w/v) L-cysteine (Solarbio, SC9730), and 0.0001% (w/v) chloramphenicol (Solarbio, R8150-1 g). The strain was incubated at 37 °C under strict anaerobic conditions for 24 to 72 h, with OD600 approximately 0.8. The bacterial suspension concentration was determined using the serial dilution method, and anaerobic cultivation and CFU counting were performed on pre-reduced agar plates. After selecting single colonies, identification was performed using 16S rRNA PCR and sequencing, and the purity of the culture was assessed by combining colony morphology and molecular verification. For AEVs isolation, *A. muciniphila* was cultured to the logarithmic phase (OD600 ≈ 0.8). The culture was centrifuged at 10,000 × g for 30 min to remove the cells, and the supernatant was filtered through a 0.22 μm filter. Then, AEVs were collected by ultracentrifugation at 170,000 × g for 60 min at 4 °C. After one wash with sterile PBS, the pellet was ultracentrifuged again, resuspended in sterile PBS, and stored at −80 °C. Nanoparticle tracking analysis (NTA) (PARTICLE METRIX, ZETAVIEW) was used to measure the particle size distribution and average size of the EVs. The PAEVs were isolated using a protocol similar to that used for AEVs. Briefly, *A. muciniphila* and its culture supernatant were pasteurized at 70 °C for 30 min. The inactivation of pasteurized *A. muciniphila* was confirmed before EV extraction as shown in Supplementary Figure 8. The supernatant was then centrifuged at 10,000 × g for 30 min, filtered through a 0.22 μm membrane, and ultracentrifuged at 170,000 × g for 60 min at 4 °C to collect PAEVs. NTA was used to determine the particle size distribution and mean particle diameter.

### 2.2 Animals and procedures

Male C57BL/6 mice (6–8 weeks old) were purchased from Beijing Huafukang Biotechnology Co., Ltd. Mice were housed at 25 °C under a 12 h light/dark cycle with free access to food and water. All animal procedures were approved by the Animal Care and Use Committee of Sichuan University (approval No. 20230412007).

After 1 week of acclimatization, the mice were randomly assigned to four groups: (1) water + PBS group (Ctrl), (2) DSS + PBS group (D-PBS), (3) DSS + AEV group (D-AEV), and (4) DSS + PAEV group (D-PAEV). IBD was induced in all model groups by administering 2% (w/v) DSS in the drinking water for 5 consecutive days, whereas the Ctrl group received regular drinking water. Starting on day 1 of DSS administration, mice were gavaged daily with 150 μL PBS or the corresponding AEV or PAEV preparation (20 μg in 150 μL PBS). Body weight, stool consistency, and fecal occult blood were monitored throughout the experiment. The disease activity index (DAI) was scored according to the criteria shown in the Supplementary Table 1. On day 7, mice were anesthetized with inhaled isoflurane (3% in oxygen). After deep anesthesia, mice were euthanized by cervical dislocation by trained personnel, and colon tissues and fresh fecal samples were collected for subsequent analyses. The animal procedures were approved by the Institutional Animal Care and Use Committee of Sichuan University (approval No. 20230412007).

### 2.3 H&E staining

The tissue samples were fixed in 4% paraformaldehyde, routinely dehydrated, cleared, embedded in paraffin, and sectioned into 3 μm serial sections. Then the sections were deparaffinized, rehydrated, stained with hematoxylin, differentiated with hydrochloric acid ethanol, blued with ammonia water, counterstained with eosin, dehydrated through a graded ethanol series, cleared, and mounted with neutral resin. Images were observed and captured under a light microscope, and related morphological quantitative analyses were performed using ImageJ software.

### 2.4 Alcian blue staining

After dewaxing, rehydration, Alcian Blue staining, Nuclear Fast Red counterstaining, gradient dehydration, clearing and mounting, histological images were captured under an optical microscope. The positive area was quantified using ImageJ software.

### 2.5 Immunofluorescence (IF) analysis

The tissue samples were fixed in 4% paraformaldehyde, routinely embedded in paraffin, and sectioned. After deparaffinization and graded rehydration, the paraffin sections were subjected to heat-induced antigen retrieval for antigen exposure, followed by blocking, primary antibody incubation, and subsequent staining according to the immunohistochemistry/ IF procedures. The antibodies used for IF detection are shown in Supplementary Table 2.

### 2.6 Western blot

Tissue samples and cultured organoids were lysed in an appropriate volume of loading buffer, boiled at 100 °C for 5 min, and then mixed with loading dye for subsequent use. The lysates were centrifuged at 12,000 rpm for 10 min, and the supernatants were collected for loading. Equal amounts of protein were separated by SDS-PAGE system, transferred onto NC membranes, and incubated with the indicated specific primary and secondary antibodies. Protein bands were detected and imaged using the LI-COR Odyssey imaging system. The antibodies used are shown in Supplementary Table 2.

### 2.7 RNA extraction and quantitative real-time PCR (qPCR)

Cells or frozen tissues were lysed with TRIzol reagent (Invitrogen, 15,596,026) to extract RNA. Then, 1 μg of RNA was reverse-transcribed into cDNA using an RT kit (Takara, RR047A). Finally, SYBR Green–based RT-PCR was performed with the CFX96 Real-Time System (Bio-Rad, Hercules, CA, USA). The primers used are shown in Supplementary Table 3.

### 2.8 Myeloperoxidase (MPO) analysis

Colon tissues were homogenized in phosphate buffer (50 mM KH₂PO₄, pH 6.0) containing 0.5% hexadecyltrimethylammonium bromide (HTAB; Sigma, H6269). After centrifugation, the supernatants were collected. An appropriate volume of tissue supernatant was then added to 200 μL of reaction buffer containing o-phenylenediamine (0.167 mg/mL; Sigma, D3252) and hydrogen peroxide (0.5 μL/mL of 1% H₂O₂; Sigma, H1009) in phosphate buffer (pH 6.0). Absorbance at 460 nm was recorded immediately at 37 °C for 30 min, with measurements taken once per minute. MPO activity was normalized to the total protein concentration determined by the BCA assay.

### 2.9 Gut microbiota profiling

To investigate how *A. muciniphila*-derived AEV and PAEV regulate intestinal homeostasis in DSS-induced IBD, fresh fecal samples were collected from each group for gut microbiota analysis, and 16S rRNA gene amplicon sequencing (NovoMagic) was performed to assess changes in fecal microbial composition. Briefly, total microbial DNA was extracted from fecal samples, and the V4 region of the bacterial 16S rRNA gene was amplified using barcoded primers (515F/806R). The PCR products were purified, quantified, pooled at equimolar concentrations, and used for library construction followed by paired-end sequencing on the Illumina platform. Raw sequencing reads were demultiplexed, primer-trimmed, merged, and quality-filtered, and chimeric sequences were identified and removed using the SILVA reference database. Amplicon sequence variants (ASVs) were then inferred using the DADA2 pipeline in QIIME2 and taxonomically assigned based on the SILVA database. To ensure comparability among samples, sequencing depth was normalized prior to downstream analyses, including alpha diversity analysis, beta diversity comparison, and ordination analysis of community structure. Data visualization was performed using the online platforms NovoMagic, Gene Cloud, and Bioinformatics.

### 2.10 Statistical analysis

Data from experiments are shown as mean ± SD. For comparisons among multiple groups, data distribution and variance homogeneity were first assessed to determine the appropriate statistical test. Normally distributed data were analyzed by one-way ANOVA, followed by appropriate *post hoc* multiple-comparison tests. For data that did not meet the assumptions of normality, the Kruskal–Wallis test was used, followed by Dunn’s post hoc test for multiple comparisons.

## 3 Results

### 3.1 Characterization of AEVs and PAEVs from *Akkermansia muciniphila*

**Figure 2:**
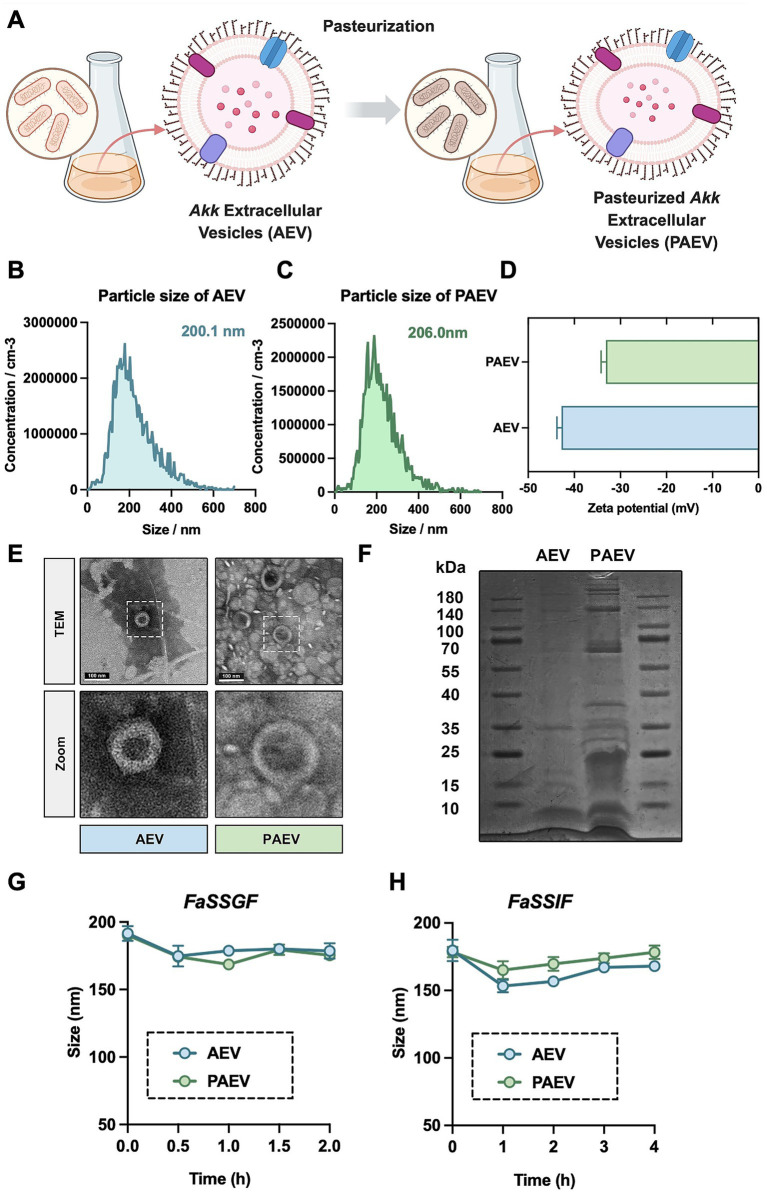
Characterization of AEVs and PAEVs. **(A)** Schematic illustration of AEVs and PAEVs. Created in BioRender. Xu, Y. (2026) https://biorender.com/qc6ouyr. **(B,C)** Particle size distribution of AEVs and PAEVs, respectively. **(D)** Zeta potential of AEVs and PAEVs. **(E)** TEM images of AEVs and PAEVs. **(F)** SDS-PAGE analysis of AEVs and PAEVs. **(G,H)** Variations in the particle sizes of AEVs and PAEVs following incubation in FaSSGF and FaSSIF for predetermined time intervals (mean ± SD; *n* = 3). Statistical significance was determined using one-way ANOVA or Student’s *t*-test, as appropriate.

Previous studies have shown that AEVs can improve intestinal barrier function and alleviate inflammation (Zheng et al., 2023), while pasteurized *A. muciniphila* also exerts protective effects in IBD models (Xue et al., 2023). However, it remains unclear whether pasteurization affects the physicochemical characteristics and biological activities of AEVs. Therefore, in this present study, natural AEVs and PAEVs were prepared, and their physicochemical properties and biological functions were comparatively analyzed (Figure 2A).

To characterize the differences between the two types of vesicles, nanoparticle tracking analysis (NTA) was used to determine particle size. The results showed that the mean diameter of AEVs was 200.1 nm, whereas that of PAEVs was 206 nm (Figures 2B,C), indicating that the two vesicle types were generally similar in size distribution. Further zeta potential analysis revealed differences in surface charge between the two vesicle types: the zeta potential of AEVs was −42.8 ± 0.99 mV, while that of PAEVs was −33.15 ± 1.06 mV (Figure 2D), suggesting that pasteurization may alter the physicochemical properties of the vesicle surface. Transmission electron microscopy (TEM) further confirmed that both AEVs and PAEVs retained a typical vesicle-like morphology, indicating that *A. muciniphila*-derived vesicles can generally maintain a relatively intact membranous structure even after pasteurization. However, compared with AEVs, PAEVs exhibited relatively less distinct vesicle boundaries and less clearly defined contours, suggesting that their membrane structure or vesicle-associated components may undergo a certain degree of rearrangement following pasteurization (Figure 2E). Furthermore, to compare the protein cargo profiles of extracellular vesicles derived from live and pasteurized *A. muciniphila*, AEVs and PAEVs were subjected to SDS-PAGE analysis. Coomassie staining revealed distinct protein banding patterns between the two EV preparations. Compared with AEVs, PAEVs displayed a more complex and intense protein profile, with prominent bands distributed across a broad molecular weight range, particularly around 25 kDa and 10–15 kDa, as well as several higher-molecular-weight bands. In contrast, AEVs showed relatively weaker protein signals, with limited detectable bands mainly around 35 kDa and the low-molecular-weight region. These results indicate that pasteurization markedly alters the protein composition or relative abundance of EV-associated proteins released into the culture supernatant, suggesting that PAEVs may contain a distinct set of protein cargo compared with conventional AEVs (Figure 2F). Notably, a prominent band at approximately 32–33 kDa was retained in both AEVs and PAEVs, which may correspond to Amuc_1100 (Plovier et al., 2017).

In addition, we further evaluated the physicochemical stability of AEVs and PAEVs in biomimetic gastrointestinal fluids. To simulate gastrointestinal transit, the samples were incubated in fasted-state simulated gastric fluid (FaSSGF) for 2 h or incubated in fasted-state simulated intestinal fluid (FaSSIF) for 4 h (Figures 2G,H). The results showed that, in both FaSSGF and FaSSIF, the particle sizes of AEVs and PAEVs changed only slightly and remained within an approximate range of 160–190 nm, indicating that both vesicle types possess good structural stability under biomimetic gastrointestinal conditions. In FaSSGF, the particle size of PAEVs at 1 h was slightly smaller than that of AEVs (PAEVs ≈ 168.6 nm; AEVs ≈ 178.7 nm), whereas the two remained generally comparable at the other time points. In FaSSIF, the particle size of AEVs was consistently slightly smaller than that of PAEVs during the 1–4 h incubation period, with a difference of approximately 10 nm. Overall, these findings indicate that both AEVs and PAEVs can withstand simulated gastrointestinal conditions without obvious aggregation or disintegration, thereby supporting their potential as oral therapies.

### 3.2 PAEV alleviated clinical symptoms and colon shortening in DSS-induced IBD

**Figure 3:**
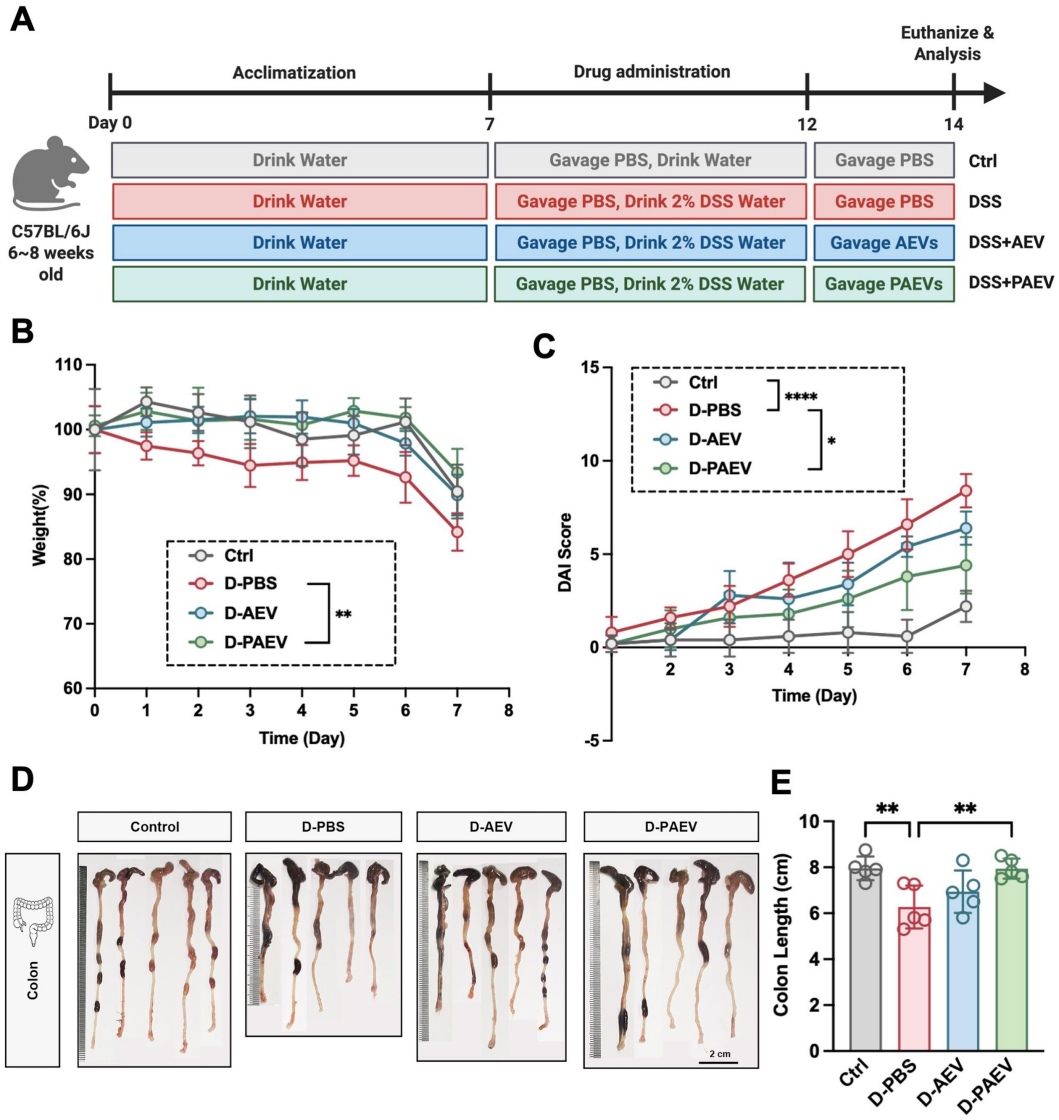
Therapeutic effects of AEV and PAEV on DSS-induced IBD. **(A)** Experimental design and grouping of animals. Created in BioRender. Xu, Y. (2026) https://BioRender.com/be3vmx5. **(B)** Body weight changes and **(C)** DAI in each group. **(D)** Representative images of the colon and **(E)** quantification of colon length (scale bar, 2 cm). Data are presented as mean ± SD (*n* = 5). Normality was checked before analysis. At the final time point, body weight and DAI were compared among groups using one-way ANOVA or the Kruskal–Wallis test, as appropriate, with *post hoc* testing. Colon length was analyzed by one-way ANOVA. *p < 0.05, **p < 0.01, ****p < 0.0001.

Based on previous evidence showing that AEV can alleviate colitis (Zheng et al., 2023; Chen et al., 2026), the present study further evaluated the protective effects of both AEVs and PAEVs in a DSS-induced acute colitis model. Specifically, acute colitis was induced by administering 2% (w/v) DSS in the drinking water. These groups were designated as D-PBS, D-AEV, and D-PAEV, respectively. Disease-related indicators, including body weight changes, stool consistency, and fecal occult blood, were continuously monitored, and the disease activity index (DAI) was calculated accordingly (Figure 3A). The results showed that, compared with the D-PBS group, PAEVs treatment significantly alleviated DSS-induced body weight loss (Figure 3B). In contrast, the D-AEV group did not show an ideal effect in improving weight loss. In addition, PAEV treatment significantly reduced the DAI. Although D-AEV group also showed a DAI-lowering effect, its efficacy was weaker than that of PAEV (Figure 3C). Further evaluation of the gross colonic phenotype after sacrifice revealed that DSS treatment caused marked colon shortening, which is one of the typical pathological features of DSS-induced colitis (Yang and Merlin, 2024). However, PAEV intervention significantly attenuated this change, whereas the D-AEV group showed no significant reduction of colon shortening (Figures 3D,E). Taken together, these results indicate that PAEV exerts a stronger protective effect than AEV in alleviating the phenotype of DSS-induced acute colitis.

### 3.3 PAEV attenuated colonic histological injury and restored goblet cell numbers

**Figure 4:**
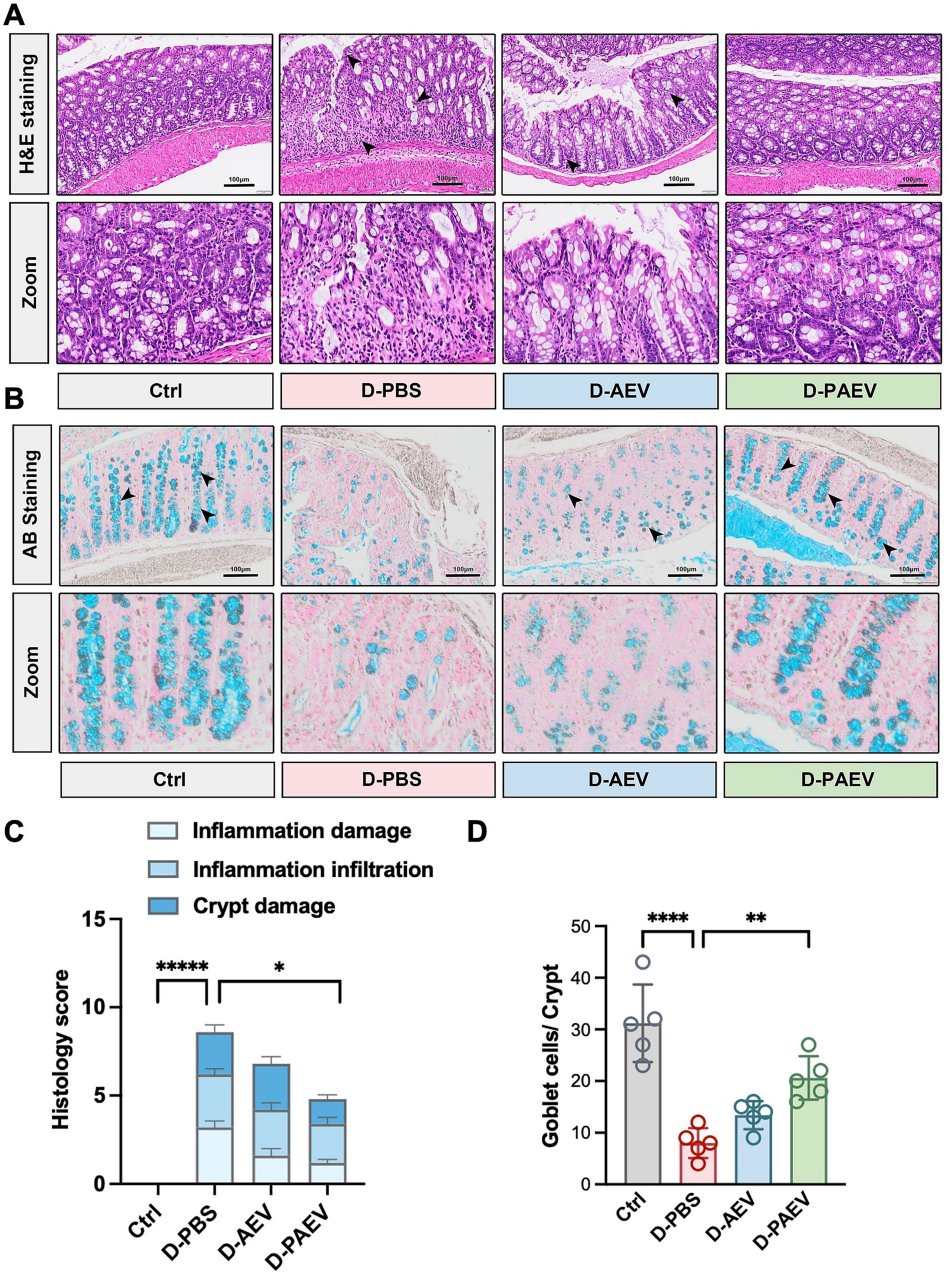
Histopathological analysis of colonic tissues. **(A)** Representative H&E staining of colon sections (scale bar, 100 μm). **(B)** AB staining of colonic tissue (scale bar, 100 μm). Quantification of histological scores for **(C)** H&E staining and **(D)** AB staining. Data are presented as mean ± SD (*n* = 5). Histological scoring was performed in a blinded manner. Statistical significance was determined using one-way ANOVA. **p < 0.01, ****p < 0.0001.

Histological analysis can further evaluate the severity of DSS-induced colitis. Hematoxylin and eosin (H&E) staining showed that DSS treatment caused marked pathological damage in the colonic tissue of mice, mainly characterized by disruption of epithelial architecture, disorganization or partial loss of crypt structure, and increased inflammatory cell infiltration, all of which are typical histological features of DSS-induced colitis (Chassaing et al., 2014; Figure 4A). Compared with the D-PBS group, both AEV and PAEV treatments alleviated colonic histological injury and reduced histological scores to varying degrees, with the improvement being more pronounced in the PAEV group (Figures 4A,C). Further Alcian blue staining revealed that DSS treatment significantly reduced goblet cell numbers and impaired mucus-related phenotypes. PAEV intervention markedly promoted goblet cell recovery, whereas the improvement in the D-AEV group was relatively limited, with no obvious difference in goblet cell number compared with the D-PBS group (Figures 4B,D). Taken together, these results indicate that both AEV and PAEV can alleviate DSS-induced colonic tissue injury, but PAEV exhibits a stronger protective effect in improving histological damage and restoring goblet cell-associated phenotypes.

### 3.4 PAEV preserves tight junction protein expression and localization in DSS-induced colitis

**Figure 5:**
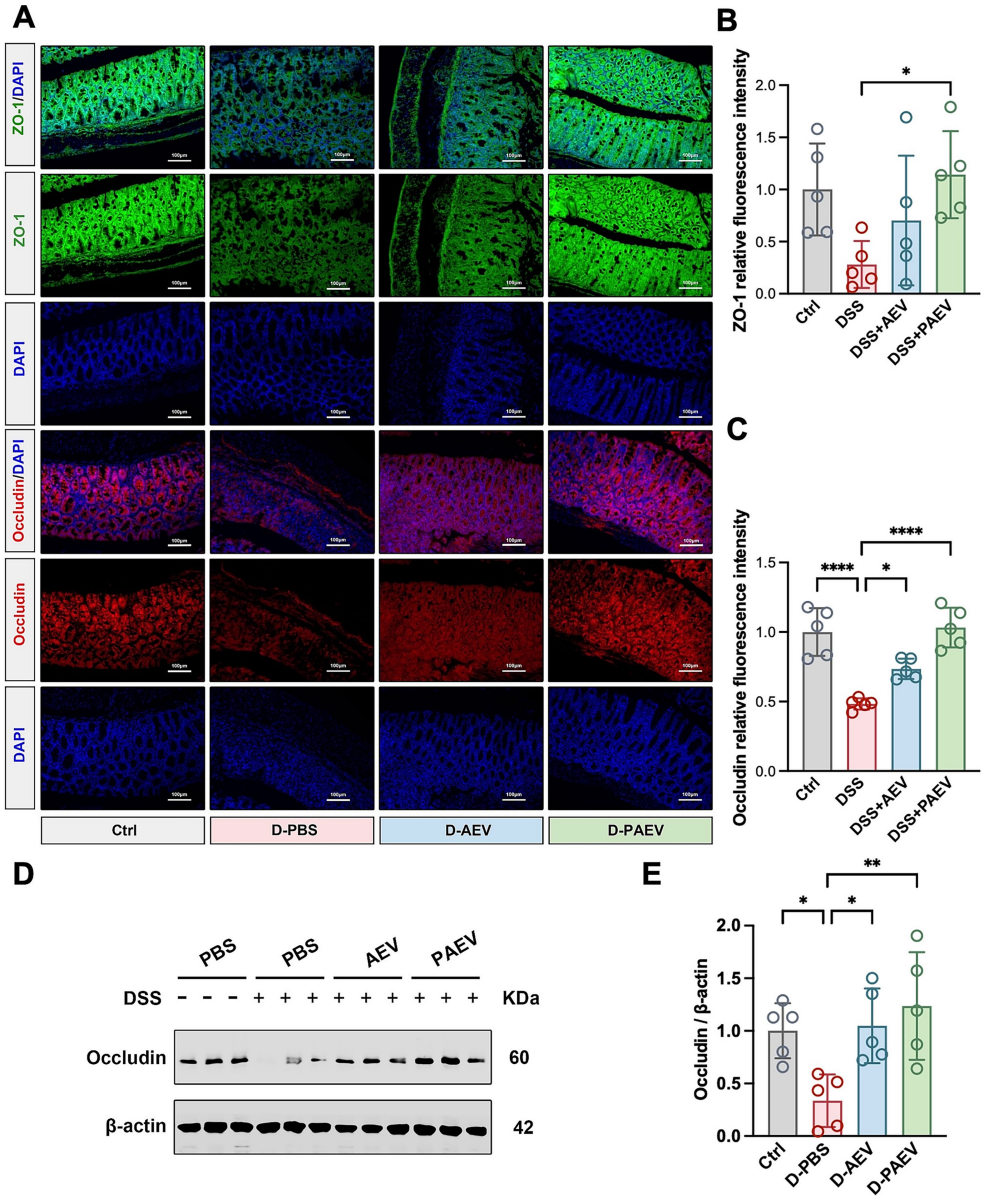
Barrier protein analysis of colonic tissues. IF staining of **(A)** ZO-1 and Occludin in colonic tissue (scale bar, 100 μm). Quantification of IF staining scores for **(B)** ZO-1 and **(C)** Occludin. Data are presented as mean ± SD (*n* = 5). **(D)** Western blot analysis of Occludin expression and **(E)** its quantification in colonic tissue. Data are presented as mean ± SD (*n* = 5). Data distribution was assessed before analysis. Group comparisons were performed using one-way ANOVA or the Kruskal–Wallis test, as appropriate, with post hoc analysis. Significance was indicated as *p < 0.05, **p < 0.01, and ****p < 0.0001. Quantification of IF was performed in a blinded manner.

Tight junction proteins constitute the key structural basis for maintaining intestinal epithelial barrier integrity and can restrict the transepithelial leakage of harmful substances and pathogens (Horowitz et al., 2023). Therefore, we further evaluated the changes in the expression and localization of ZO-1 and Occludin (Figures 5A–C). Immunofluorescence (IF) analysis showed that DSS treatment disrupted the continuity of ZO-1 and Occludin at epithelial cell borders and reduced their expression, whereas PAEV treatment significantly restored their continuous distribution at the membrane/cell junctions; in contrast, no obvious improvement was observed in the AEV group (Figures 5A–C). Consistent with these findings, Western blot analysis showed that DSS reduced the protein level of Occludin, whereas PAEV and AEV treatment significantly upregulated and partially restored its expression (Figures 5D,E). Taken together, these results indicate that, under the conditions of the present study, PAEV improved both the junctional localization of ZO-1 and Occludin protein levels, whereas AEV improved only Occludin expression and had no apparent effect on ZO-1 expression.

These results indicate that PAEV treatment partially restored DSS-disrupted tight junction features, including the junctional localization of ZO-1 and Occludin, as well as Occludin protein expression. In contrast, AEV treatment exerted only limited effects on these indicators under the present experimental conditions. These findings suggest that PAEV more effectively preserves tight junction integrity in DSS-induced colitis, although the precise signaling mechanisms through which PAEV regulates tight junction protein expression or localization require further investigation.

### 3.5 PAEV partially attenuates DSS-induced colonic inflammatory responses

**Figure 6:**
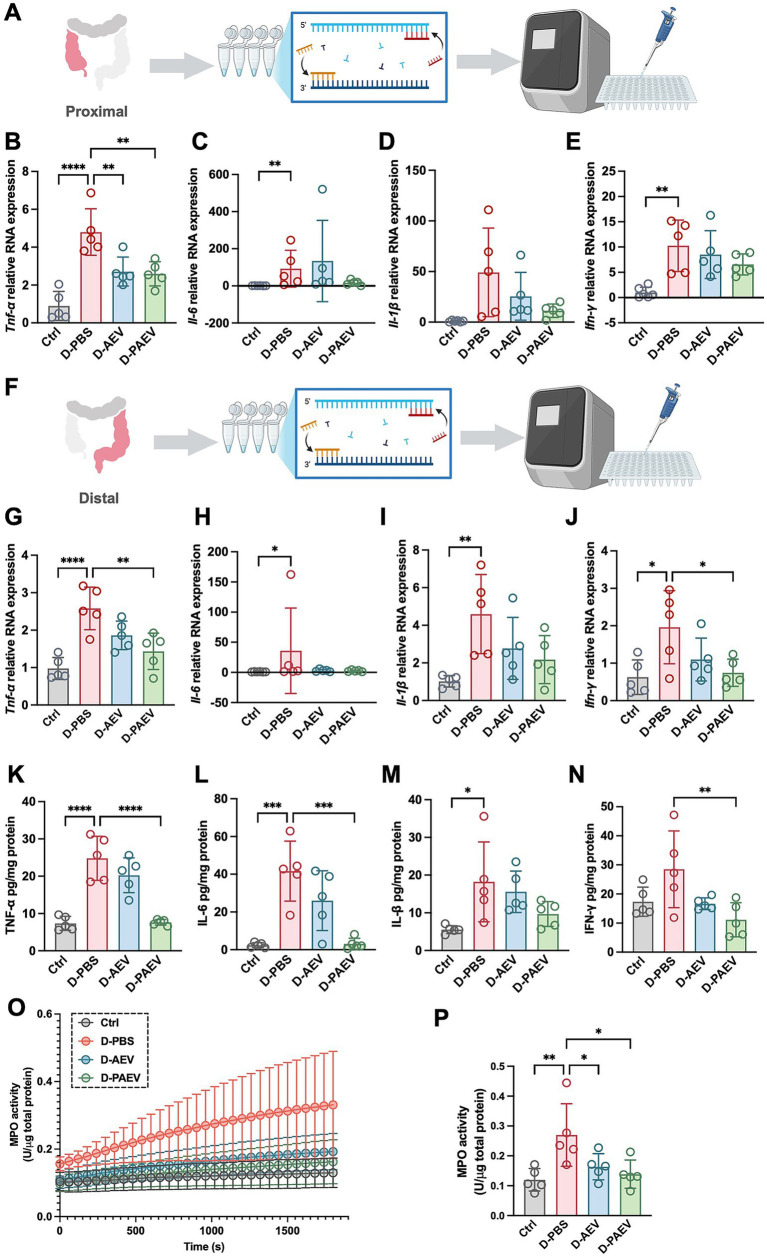
Inflammatory cytokine levels in colonic tissues. **(A–E)** mRNA expression levels of inflammatory cytokines in the proximal colon. **(F–J)** mRNA expression levels of inflammatory cytokines in the distal colon. **(K–N)** Protein levels of inflammatory cytokines in colon tissue. **(O,P)** MPO activity kinetics in colon tissue over 30 min, with a representative measurement shown at 960 s. Data are presented as the mean ± SD (*n* = 5). Data normality was verified before analysis. Group differences were examined using one-way ANOVA or Kruskal–Wallis test with corresponding post-hoc tests. **(A)** and **(F)** were created in BioRender. Xu, Y. (2026) https://BioRender.com/gqf3ezc. *p < 0.05, **p < 0.01, ***p < 0.001, ****p < 0.0001.

The levels of pro-inflammatory factors in colonic tissue are important indicators for characterizing colitis (Neurath, 2024). To further evaluate the effects of AEV and PAEV on local inflammatory responses in DSS-induced colitis, we measured the mRNA expression levels of inflammatory cytokines in the colon (Figures 6A–J). The results showed that DSS treatment significantly upregulated *Tnf-α* mRNA expression in the proximal colon. Both AEV and PAEV interventions markedly reduced *Tnf-α* mRNA levels (Figure 6B). In addition, DSS treatment significantly upregulated *Il-6* mRNA expression compared with the Ctrl group. While D-AEV treatment did not significantly reduce *Il-6* expression, D-PAEV treatment showed a trend toward decreased *Il-6* mRNA expression, approaching levels observed in controls, although this reduction did not reach significant difference relative to the D-PBS group (Figure 6C). Furthermore, *Il-1β* showed an upward trend in the D-PBS group compared with the Ctrl group, the differences did not reach statistical significance (Figure 6D). Moreover, DSS treatment significantly induced *Ifn-γ* expression, whereas both AEV and PAEV treatment showed a downward trend compared with the D-PBS group, although the differences did not reach statistical significance (Figure 6E). In the distal colon, inflammatory changes were more pronounced than those in the proximal colon (Figures 6F–J). Compared with the Ctrl group, the mRNA levels of *Tnf-α*, *Il-6, Il-1β*, and *Ifn-γ* were all significantly elevated in the D-PBS group. Although *Tnf-α* expression showed a reduced trend following AEV treatment, the change was not statistically significant, whereas PAEV treatment resulted in a significant reduction. Both AEV and PAEV treatments led to a downward trend in *Il-6* mRNA levels; however, these differences did not reach statistical significance. *Il-1β* showed a downward trend after both AEV and PAEV treatment, with lower levels in the D-PAEV group, but the differences did not reach statistical significance compared with the D-PBS group. *Ifn-γ* expression exhibited a modest reduction following AEV treatment, whereas PAEV treatment induced a more pronounced and statistically significant decrease. Overall, PAEV exerted a stronger inhibitory effect on selected inflammatory transcripts in the distal colon, particularly *Tnf-α* and *Ifn-γ* (Figures 6G–J).

At the protein level, TNF-α, IL-6, and IL-1β in colonic tissues were all generally higher in the D-PBS group than in the Ctrl group. After AEV treatment, TNF-α levels exhibited a reduced trend, but the difference was not statistically significant, whereas PAEV treatment significantly lowered TNF-α levels compared with the D-PBS group. IL-6 protein also showed a downward trend in the AEV group, but the difference did not reach statistical significance; by contrast, the PAEV group showed a significant decrease compared with the D-PBS group. IL-1β protein levels showed a reduced trend to varying degrees after both AEV and PAEV intervention, with a lower mean value in the PAEV group, although no significant difference versus the D-PBS group was indicated. For IFN-γ protein, PAEV treatment significantly decreased its level, whereas the AEV group showed no statistically significant difference compared with the D-PBS group (Figures 6K–N). Collectively, PAEV significantly suppressed TNF-*α*, IL-6, and IFN-*γ* levels, whereas AEV did not show statistically significant effects on TNF-α, IL-6, or IFN-γ. These findings suggest that PAEV attenuates selected local inflammatory markers in DSS-induced colitis.

The MPO activity assay further supported these findings. Elevated MPO activity indicates increased neutrophil infiltration in colonic tissue and is closely associated with aggravated local inflammatory responses (Hanning et al., 2023). The dynamic MPO curve showed that MPO activity in the D-PBS group remained at the highest level throughout the entire detection period, exceeding that of the other groups. Both the D-AEV and D-PAEV groups exhibited lower MPO activity than the D-PBS group, with the D-PAEV group being overall closer to the Ctrl group. Quantitative analysis at 960 s showed that MPO activity in the D-PBS group was significantly higher than that in the Ctrl group. Both AEV and PAEV treatment significantly decreased MPO activity compared with the D-PBS group. PAEV showed a lower trend compared to the AEV group. These results indicated that AEV and PAEV were effective in reducing inflammatory cell infiltration and alleviating colonic inflammation (Figures 6O,P).

Taken together, DSS treatment induced marked inflammatory responses in the colon, as evidenced by increased expression of selected inflammatory transcripts, elevated cytokine protein levels, and enhanced MPO activity. AEV showed limited effects or trends toward reduction in several inflammatory markers, whereas PAEV significantly reduced distal colonic *Tnf-α* and *Ifn-γ* mRNA expression, lowered colonic TNF-α, IL-6, and IFN-γ protein levels, and decreased MPO activity. These findings suggest that PAEV attenuates selected local inflammatory readouts in DSS-induced colitis, although not all inflammatory markers were significantly altered.

### 3.6 PAEV mitigates DSS-induced IBD by attenuating STING-associated inflammatory responses

**Figure 7:**
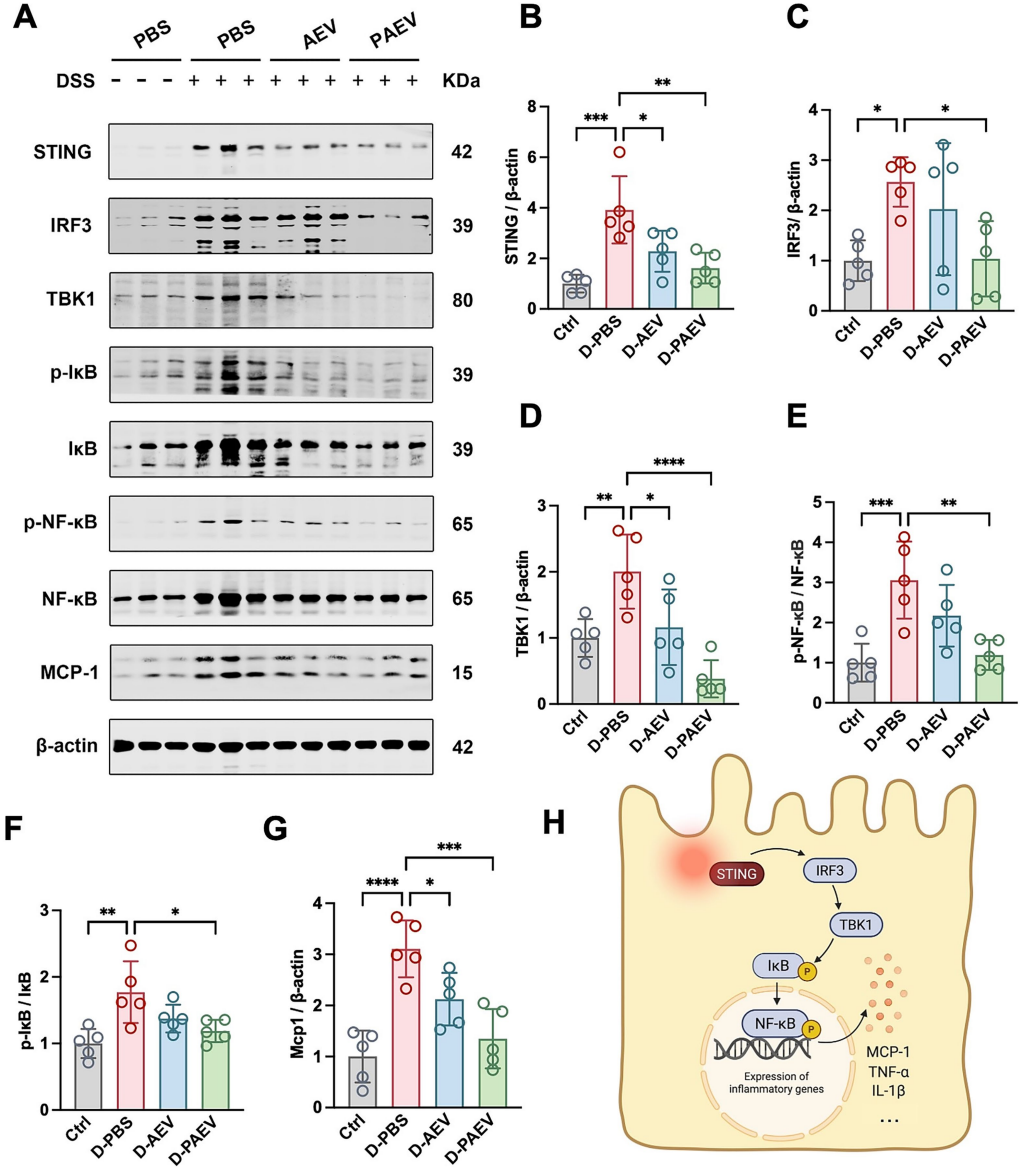
Effects of AEV and PAEV on the STING signaling axis in DSS-induced IBD. **(A)** Representative Western blot images showing the expression of STING, TBK1, IRF3, p-IκB, IκB, NF-κB, and p-NF-κB in colonic tissues from mice with DSS-induced IBD. Created in BioRender. Xu, Y. (2026) https://BioRender.com/is0opyq. **(B–G)** Quantitative analysis of the protein levels shown in panel A. Data are presented as mean ± SD (*n* = 5). Data normality was assessed before analysis. One-way ANOVA with Tukey’s test was used for normally distributed data, and Kruskal–Wallis test with Dunn’s test for non-normally distributed data. *p < 0.05, **p < 0.01, ***p < 0.001, ****p < 0.0001. **(H)** Schematic illustration of STING pathway.

Although PAEV effectively reduced the expression of proinflammatory cytokines in DSS-induced colitis, the upstream mechanisms responsible for this protective effect remained unclear. Persistently high levels of STING activation in the wrong compartment have been reported to promote epithelial injury, myeloid inflammation, pyroptosis, and colitis exacerbation (Zhang et al., 2023; Dimitrov et al., 2025). Given that the STING signaling pathway plays an important role in IBD disease processes, we determined whether AEV and PAEV participate in regulating the STING signaling pathway in DSS-induced colitis. We examined the expression and activation status of proteins related to the STING-TBK1-IRF3/NF-κB signaling axis in colonic tissues, including STING, TBK1, IRF3, p-IκB, IκB, NF-κB, and p-NF-κB. Western blot analysis showed that DSS treatment significantly upregulated the expression of STING and TBK1, whereas both AEV and PAEV interventions suppressed these changes, with PAEV showing a more pronounced inhibitory effect (Figures 7A,B,D). In addition, DSS markedly increased IRF3 expression, which was significantly reduced by PAEV treatment, while no significant change was observed in the AEV group (Figures 7A,C). DSS stimulation significantly enhanced the phosphorylation levels of IκB and NF-κB. Notably, PAEV intervention markedly inhibited these changes, whereas AEV treatment did not produce a significant improvement (Figures 7A,E,F). Further analysis showed that DSS exposure also significantly elevated the expression of the inflammation-related molecule MCP-1, while both AEV and PAEV reduced its level, with PAEV exerting a stronger inhibitory effect (Figures 7A,G). Taken together, these results indicate that both AEV and PAEV alleviate DSS-induced intestinal inflammation, at least in part, by suppressing activation of the STING-TBK1-IRF3/NF-κB signaling pathway, with PAEV exhibiting a stronger inhibitory effect (Figure 7H).

### 3.7 PAEV treatment is associated with altered gut microbiome composition

**Figure 8:**
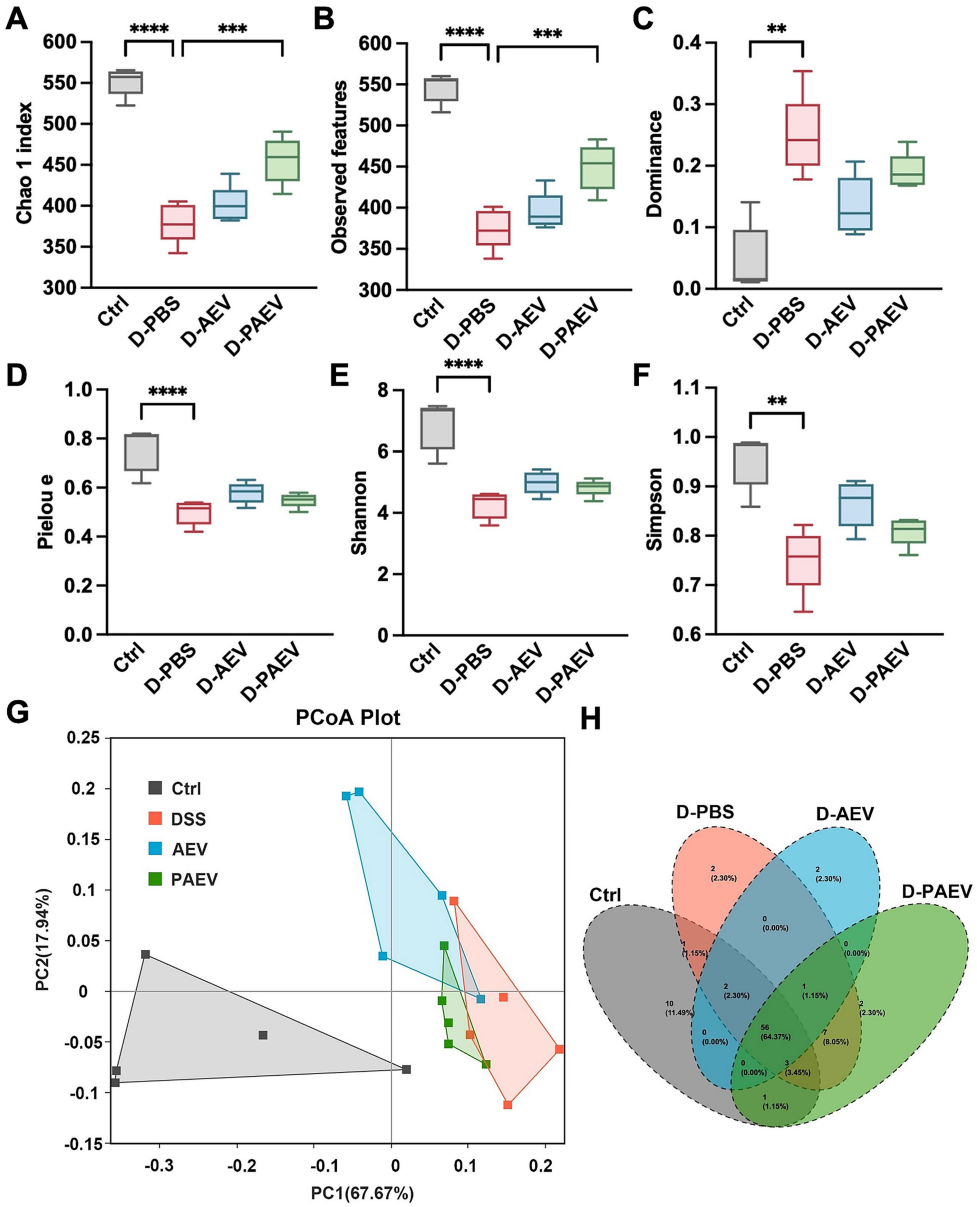
PAEV restore gut microbial diversity and community structure in DSS-induced IBD mice. **(A–F)** Alpha-diversity indices (Chao1, dominance, observed features, Pielou’s evenness, Shannon, and Simpson). Data are presented as min to max (*n* = 5). Normality was assessed before analysis. Normally distributed data were analyzed by one-way ANOVA with Tukey’s test, whereas non-normal data were analyzed by Kruskal–Wallis with Dunn’s test. **p < 0.01, ***p < 0.001, ****p < 0.0001. **(G)** PCoA plot showing clustering of gut microbial communities. **(H)** Venn diagram of shared and unique microbial features among groups.

To elucidate the regulatory effects of AEV and PAEV administered orally on the gut microbiota of DSS-induced IBD mice, we performed 16S rRNA gene sequencing on colonic fecal samples from the Ctrl, D-PBS, D-AEV, and D-PAEV groups. The results of the Alpha-diversity of the gut microbiota showed that compared to the Ctrl group, the Chao1 index and Observed features in the D-PBS group were significantly reduced (Figures 8A,B; Supplementary Figures S4A,B), indicating that DSS treatment significantly weakened the microbial richness. Compared to the D-PBS group, D-PAEV group exhibited significantly elevated Chao1 index and Observed features, suggesting that PAEV effectively restores the DSS-induced reduction in microbial richness. In contrast, the D-AEV group showed only a slight upward trend. Further analysis showed that DSS treatment leads to a decrease in Dominance index, Pielou’s evenness, Shannon index, and Simpson index (Figures 8C–F; Supplementary Figures S4C–F), indicating that microbial evenness and overall diversity are impaired. Although both AEV and PAEV showed partial upward trends in these diversity-related indices compared with D-PBS, the differences did not reach statistical significance. Collectively, these results indicate that PAEV effectively restores DSS-induced loss of gut microbial richness, whereas neither PAEV nor AEV significantly improves microbial evenness or overall diversity.

To further assess the effects of AEV and PAEV on DSS-induced gut microbiota dysbiosis, we analyzed microbial beta diversity and the shared or group-specific microbial features. PCoA revealed a clear separation between the Ctrl and D-PBS groups (PC1 = 67.67%, PC2 = 17.94%) (Figure 8G), indicating that DSS markedly altered the overall gut microbial community structure. Compared with the D-PBS group, both AEV and PAEV reshaped the microbial composition, although with distinct trajectories. Notably, the D-AEV group showed greater intra-group dispersion, suggesting variable effects across individuals, whereas the D-PAEV group clustered more tightly and was clearly separated from the D-PBS group. Further Venn diagram analysis shows that there are 56 shared microbial features among the four groups, accounting for 64.37% of the total features (Figure 8H), indicating that a large core microbiome composition is still retained among the groups. It is worth noting that the number of specific microbial features in the Ctrl group was 10 (11.49%), while the D-PBS group had only 2 (2.30%), suggesting that DSS treatment may lead to the loss of some normal-associated microbial features. At the same time, both the D-AEV group and the D-PAEV group retained their respective specific microbiome characteristics and exhibited varying degrees of overlap with other groups, indicating that both types of vesicle interventions not only affect the relative composition of the core microbiome but may also promote the formation of certain treatment-related microbiome characteristics.

**Figure 9:**
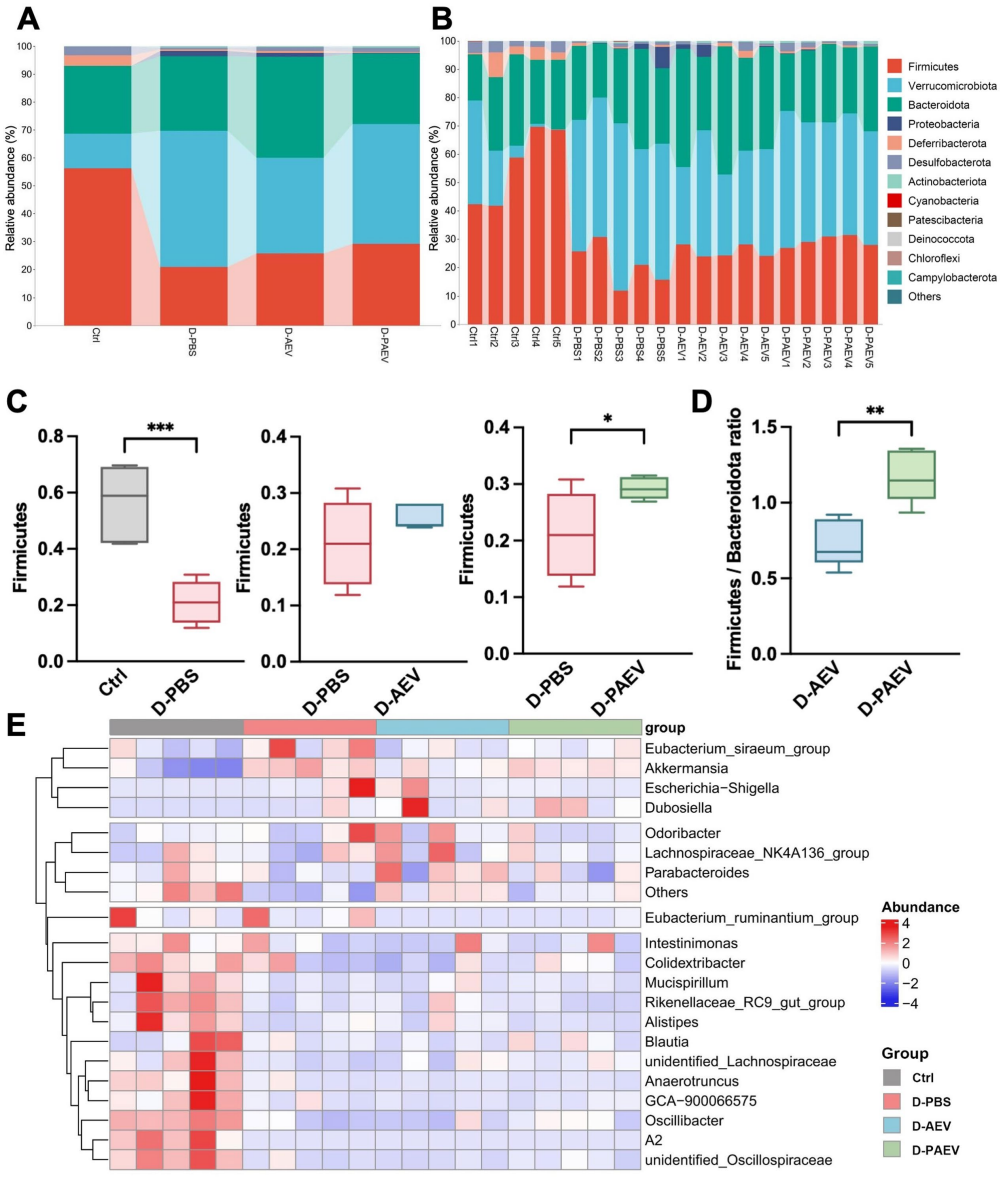
PAEV reshapes gut microbial composition in DSS-induced IBD mice. **(A,B)** Stacked bar plots showing taxonomic composition at the phylum level. **(C,D)** Relative abundances of **(C)** *Firmicutes* and **(D)** the *Firmicutes/Bacteroidota* ratio. Data are presented as min to max (*n* = 5). Normally distributed data were analyzed using an unpaired two-tailed Student’s *t*-test, whereas non-normally distributed data were analyzed using the Mann–Whitney U test. *p < 0.05, **p < 0.01, ***p < 0.001. **(E)** Heatmap of genus-level relative abundances of gut microbiota.

To further clarify the impact of AEV and PAEV on DSS-induced gut microbiota structural abnormalities, we analyzed the compositional changes of the microbiota at the phylum and genus levels. The stacked bar chart at the phylum level shows that the intestinal microbiota in the Ctrl group is mainly composed of *Firmicutes*, *Verrucomicrobiota*, and *Bacteroidota*, while the overall composition of the microbiota is reshaped after DSS treatment, with the most notable change being a significant decrease in the relative abundance of *Firmicutes* (Figures 9A,B). *Firmicutes* abundance was positively correlated with colon length and negatively correlated with pro-inflammatory markers (Supplementary Figure S5). DSS treatment markedly altered the gut microbial composition, as shown by the distinct microbial profiles among the control, DSS-treated, AEV-treated, and PAEV-treated groups. DSS exposure significantly reduced the relative abundance of Firmicutes compared with the control group. Notably, PAEV treatment partially restored the abundance of Firmicutes, whereas AEV treatment showed a weaker effect (Figure 9C). In addition, PAEV administration significantly increased the Firmicutes/Bacteroidota ratio compared with AEV treatment (Figure 9D). These findings suggest that PAEVs may alleviate DSS-induced gut microbial dysbiosis by promoting a shift toward a Firmicutes-enriched microbial profile.

To further analyze the impact of DSS-induced IBD and the intervention of AEV and PAEV on the gut microbiota, we created heatmaps of the differential abundance taxa at the genus level and conducted hierarchical clustering analysis. The results showed that DSS treatment significantly reshaped the composition of the gut microbiota, and different genera responded differently to AEV and PAEV. Specifically, the enrichment of the *Escherichia-Shigella* genus can disrupt the intestinal barrier and activate inflammation, making it an important factor in the occurrence and progression of IBD (Wang et al., 2024). Indeed, *Escherichia-Shigella* were negatively correlated with colon length and positively correlated with pro-inflammatory markers, particularly TNF-*α* and IL-1β, suggesting their association with aggravated colonic inflammation from Spearman correlation analysis (Supplementary Figure S6). *Escherichia-Shigella* showed an increasing trend in the D-PBS group, while its abundance tended to decrease after AEV and PAEV treatment, particularly in the D-PAEV group; however, these differences did not reach statistical significance (Figure 9E; Supplementary Figure S7). In addition, *Blautia* is generally considered a symbiotic bacterium associated with the maintenance of intestinal homeostasis, with potential protective effects against IBD (Liu et al., 2021). Moreover, *Blautia* showed a decreasing trend after DSS treatment and a mild recovery trend following AEV or PAEV intervention, although no significant differences were observed among groups (Figure 9E; Supplementary Figure S7B). The above results suggest that DSS-induced IBD may be related to the imbalance of certain key bacterial genera, and that AEV and PAEV, particularly PAEV, may have some regulatory potential for this dysbiosis, although most genus-level differences did not reach statistical significance.

## 4 Discussion

Previous studies have extensively demonstrated the beneficial effects of live *A. muciniphila*, pasteurized *A. muciniphila*, and extracellular vesicles derived from *A. muciniphila* in intestinal and metabolic diseases. However, most existing studies have focused on the whole bacterium or extracellular vesicles released by live bacteria, leaving it largely unclear whether pasteurization functionally modifies *A. muciniphila*-derived vesicles and enhances their therapeutic potential in IBD. A growing body of evidence supports the beneficial role of *A. muciniphila* in preserving intestinal homeostasis, yet the therapeutic use of live bacteria in IBD remains controversial because bacterial viability may lead to variable host responses under different inflammatory or ecological conditions (Ansaldo et al., 2019). Therefore, the novelty of the present study lies in the systematic comparison of extracellular vesicles derived from both live and pasteurized *A. muciniphila* in IBD treatment. In the present study, we demonstrate that extracellular vesicles derived from pasteurized *A. muciniphila* exert pronounced protective effects in DSS-induced colitis and outperform vesicles derived from the live bacterium. Specifically, PAEVs significantly attenuated body weight loss, improved histopathological injury, reduced pro-inflammatory cytokines, and restored epithelial barrier integrity through upregulation of tight junction proteins. By contrast, AEVs showed only limited efficacy, mainly reflected by partial improvement in some of the tight junction proteins and inflammatory cytokines. These findings suggest that PAEVs are functionally distinct from AEVs and may possess enhanced anti-inflammatory and barrier-protective activity.

The superior efficacy of PAEVs is particularly noteworthy in the context of IBD, a disorder characterized by persistent mucosal inflammation and epithelial barrier dysfunction. Impaired barrier integrity facilitates the translocation of luminal antigens and microbial products into the mucosa, thereby amplifying immune activation and sustaining chronic inflammation. In this study, PAEVs markedly enhanced the expression of tight junction-related proteins, indicating improved epithelial integrity. This effect is of particular biological relevance because reinforcement of the intestinal barrier may interrupt the feed-forward cycle between epithelial damage and inflammatory signaling. The weaker response observed with AEVs further raises the possibility that vesicles derived from pasteurized bacteria may possess a more favorable functional composition, allowing them to exert stronger protective effects under inflammatory conditions. Thus, compared with conventional AEVs, PAEVs may represent a refined postbiotic strategy that retains the advantages of microbial-derived vesicles while avoiding potential risks associated with live bacterial administration.

As a potential mechanism, reduced activation of the STING/IκB/NF-κB pathway may underlie the protective effects of PAEVs. STING-related signaling has been implicated in colitis-associated inflammatory responses, with activation of the IκB/NF-κB axis promoting expression of pro-inflammatory mediators involved in intestinal injury (Mukherjee et al., 2024). In our study, PAEV treatment correlated with diminished activation of this signaling cascade, along with decreases in selected inflammatory cytokines and improvements in barrier-related markers. Future studies utilizing STING-deficient models, pathway-specific inhibitors, or rescue strategies will be essential to determine whether suppression of this axis is necessary or sufficient for the protective effects of PAEVs. Additionally, given that cGAS is a major upstream sensor activating STING signaling in response to cytosolic DNA, future investigation is warranted to explore whether PAEVs inhibit cGAS activation or the cGAS-dependent STING pathway in colitis.

Beyond modulating inflammatory signaling and enhancing barrier protection, PAEV treatment also appears to modulate the gut microbiota. Previous studies have linked the onset and progression of IBD to reduced gut microbiota diversity (Pinho et al., 2026), suggesting a possible role for microbiota modulation in PAEV-mediated protection, although this remains to be confirmed. In the present study, PAEV treatment increased gut microbiota diversity and partially reversed DSS-induced dysbiosis, notably improving the abundance of the beneficial phylum Firmicutes. There was also a tendency toward enrichment of certain beneficial bacterial genera alongside a reduction in potentially harmful genera. However, most of these genus-level changes were relatively modest compared to the model group. Thus, the current findings suggest that PAEVs treatment may be associated with partial modulation of DSS-induced alterations in the gut microbiota. It is also important to emphasize that the observed microbial shifts may represent secondary effects of reduced inflammation rather than direct drivers of the therapeutic efficacy of PAEVs.

However, this study has several limitations. First, while we compared the efficacy of PAEVs and AEVs at a defined dose, we did not assess dose-dependent effects. Future studies are needed to systematically evaluate the efficacy, safety, and potential toxicity of PAEVs across a range of doses. Such work will be essential to fully characterize the dose–response relationships and optimize therapeutic strategies. In addition, our results showed some differences between AEVs and PAEVs in treating acute DSS-induced colitis. After pasteurization, some of the beneficial effects of AEVs appeared to be enhanced in PAEVs, as shown by several disease indicators. The specific vesicular components responsible for these enhanced effects remain undefined. Given the limitations of current filtration- and ultracentrifugation-based isolation strategies, we cannot completely rule out the possibility that other bacterial components, soluble proteins, membrane fragments, or pasteurization-derived debris were co-isolated and contributed to the observed protective effects. Future investigations should identify which proteins, lipids, polysaccharides, or nucleic acids in AEVs are altered or inactivated during pasteurization, and clarify how these changes contribute to the superior therapeutic efficacy of PAEVs. Comprehensive proteomic, lipidomic or nucleic acid profiling of AEVs and PAEVs would help to clarify this result. Another limitation is that the causal relationship between microbiota remodeling and host anti-inflammatory effects requires further investigation. Furthermore, because the present findings were obtained in an acute DSS-induced colitis model, they cannot fully reflect the chronic, relapsing nature of human IBD. This study, therefore, does not address the long-term efficacy, safety, durability of barrier repair, or relapse-preventive potential of PAEV treatment. Future studies using chronic or relapsing colitis models are needed to further evaluate the translational potential of PAEVs.

## Data Availability

The raw data generated in this study can be found in the NCBI (https://www.ncbi.nlm.nih.gov/), accession PRJNA1455147.
