# Supplementary material for: Extracellular vesicles from pasteurized Akkermansia muciniphila ameliorate inflammatory bowel disease through suppression of STING-driven inflammatory signaling
Source: Front Microbiol. 2026 Jul 22;17:1876636. doi: 10.3389/fmicb.2026.1876636 (PMC13437525; doi:10.3389/fmicb.2026.1876636)
Supplement: Supplementary file 1 [file Supplementary_file_1.DOCX]

Supplementary Materials for

**Extracellular vesicles from pasteurized *Akkermansia muciniphila* ameliorate inflammatory bowel disease through suppression of STING-driven inflammatory signalling**

Ling Zou^a^, Zhilong Jia^b^, Yutong Shu^b^, Xiang You^b^, Jingjing Ma^b^, Yu Wang^a^, Jinhan He^a,b^, Yining Xu^a, *^

*Corresponding author and emails: Prof. Yining Xu yiningxu@scu.edu.cn

**This file includes:**

Figs. S1 to S8, Table 1-3

**Supplementary Fig. 1.** Representative macroscopic images of colons with scale ruler in each group (n = 5)

**Supplementary Fig. 2.** Original Western blots (n = 5)

**Supplementary Fig. 3.** Original Western blots (n = 5).

**Supplementary Fig. 4.** Species abundance curves of Chao1, dominance, observed features, Pielou’s evenness, Shannon, and Simpson (n = 5).

**Supplementary Figure 5.** Correlation analysis of the phylum-level relative abundances of gut microbiota and IBD phenotype. (n = 5).

**Supplementary Figure 6.** Correlation analysis between genus-level gut microbiota relative abundance and the IBD phenotype. (n = 5).

**Supplementary Figure 7.** Genus-level relative abundances of gut microbiota. (n=5).

**Supplementary Figure 8.** Representative images of live *A. muciniphila* and pasteurized *A. muciniphila*.

**Supplementary Table 1.** DAI score table.

| Score | Body weight loss | Stool consistency | Fecal blood |
| --- | --- | --- | --- |
| 0 | No loss | Normal | Negative |
| 1 | 1–5% | Pastelike | Occult blood positive |
| 2 | 5–10% | Loose stool | Mild visible blood |
| 3 | 10–20% | Watery stool | Gross bleeding |
| 4 | >20% | - | - |

DAI score was performed in a blinded manner.

**Supplementary Table 2.** Primary antibodies.

| Antibody | Dilution | Catalog No. | Supplier |
| --- | --- | --- | --- |
| Rabbit-β-actin | 1:10,000 for WB | AC026 | Abclonal |
| Rabbit-Occludin | 1:2,000 for WB; 1:100 for IF | E6B4R | Cell Signaling Technology |
| Rabbit-Phospho-NF-κB p65 (Ser536) | 1:2,000 for WB | 3033 | Cell Signaling Technology |
| Rabbit-NF-κB | 1:2,000 for WB | 8242 | Cell Signaling Technology |
| Rabbit-STING | 1:2,000 for WB | 13647S | Cell Signaling Technology |
| Rabbit-IRF3 | 1:2,000 for WB | 11312-1-AP | Proteintech |
| Rabbit-TBK1 | 1:2,000 for WB | 28397-1-AP | Proteintech |
| Rabbit-p-IκB | 1:2,000 for WB | 9246S | Cell Signaling Technology |
| Rabbit-IκB | 1:2,000 for WB | 4812S | Cell Signaling Technology |
| Rabbit-MCP-1 | 1:2,000 for WB | 2029S | Cell Signaling Technology |
| Rabbit-ZO-1 | 1:100 for IF | ab96587 | Abcam |

**Supplementary Table 3.** Primer sequence.

| Name of the genes | Sequence (5’ to 3’) |  |
| --- | --- | --- |
| *mTnf-α* | For | CAGGCGGTGCCTATGTCTC |
|  | Rev | CGATCACCCCGAAGTTCAGTAG |
| *mIl-6* | For | GTCCTTCCTACCCCAATTTCCA |
|  | Rev | TAACGCACTAGGTTTGCCGA |
| *mIl-1β* | For | GCAACTGTTCCTGAACTCAACT |
|  | Rev | ATCTTTTGGGGTCCGTCAACT |
| *m18s* | For | TTGACTCAACACGGGAAACC |
|  | Rev | AGACAAATCGCTCCACCAAC |
| *mIFN-γ* | For | CAGCAACAGCAAGGCGAAAAAGG |
|  | Rev | TTTCCGCTTCCTGAGGCTGGAT |
